# Cerebellar High Frequency Transcranial Pulsed Current Stimulation Increases Total Sleep Time in Chronic Insomnia: A Pilot Study

**DOI:** 10.21203/rs.3.rs-10564909/v1

**Published:** 2026-09-18

**Authors:** Fengyi Hao, Sandra Zhong, Roger C.M. Ho, Esra Neufeld, Niels Kuster, Fariba Karimi, Jinyi Li, Xiyu Wu, Alvaro Pascual-Leone, Xiaojiang Jiang

**Affiliations:** Chongqing Normal University; King’s College London; Hong Kong University of Science and Technology; Foundation for Research on Information Technologies in Society (IT’IS); Foundation for Research on Information Technologies in Society (IT’IS); Foundation for Research on Information Technologies in Society (IT’IS); Army Medical University; Chongqing Western Hospital; Hebrew SeniorLife; Chongqing Western Hospital

**Keywords:** high frequency transcranial pulsed current stimulation, chronic insomnia, actigraphy total sleep time, cerebellar stimulation, anxiety, depression

## Abstract

Chronic insomnia is characterized by reduced sleep duration and is associated with substantial physical and psychological morbidity. Existing treatments are limited by accessibility, tolerability, and persistent residual symptoms. Given emerging evidence implicating the cerebellum in sleep–wake regulation, we evaluated the safety and efficacy of cerebellar high-frequency transcranial pulsed current stimulation (tPCS) in individuals with chronic insomnia. In this double-blind, randomized, sham-controlled pilot trial, thirty adults on stable pharmacotherapy were assigned 1: 1 to receive 12 sessions of bilateral cerebellar active tPCS (400 Hz, 0.6 mA average current, 30 minutes/session) or sham stimulation over two weeks. The primary outcome was actigraphy-derived total sleep time (TST). Between-group comparisons were performed using rank analysis of covariance adjusting for baseline values. Compared with sham stimulation, active tPCS significantly increased TST (adjusted mean difference, 34.25 minutes; 95% CI, 1.60–66.89; *P* = 0.022; partial η^2^ = 0.180) and improved Pittsburgh Sleep Quality Index (PSQI) global score, including subjective sleep quality and sleep duration. Exploratory analyses demonstrated greater reductions in anxiety and depressive symptoms. No serious adverse events occurred. These findings suggest that cerebellar high-frequency tPCS may improve objective sleep duration and subjective sleep quality in insomnia and warrant confirmation in larger randomized controlled trials.

**Trial Registration:**

Chinese Clinical Trial Registry ChiCTR2300072403, Date of registration: Jun 12, 2023; (URL: https://www.chictr.org.cn/showproj.html?proj=199226)

## Introduction

Insomnia is the most prevalent sleep disorder in the general population, affecting up to 30% of adults worldwide ^1^. Insomnia is generally defined as difficulty initiating or maintaining sleep, or waking earlier than desired, despite adequate opportunity for sleep, and associated with excessive daytime impairment ^2^. If symptoms persist for more than 3 months, insomnia is considered chronic, and it is associated with a wide range of adverse health outcomes, including an increased risk of cardiovascular disease, neuropsychiatric disorders, and an elevated all-cause mortality ^3-5^.

The clinical diagnosis of insomnia is primarily based on the patient’s subjective experience of persistent sleep difficulties and associated daytime impairment, rather than on objectively measured sleep duration. Thus, a patient may meet diagnostic criteria for insomnia disorder despite having a normal sleep duration on objective measures such as actigraphy. However, it is commonly assumed that there is a presence of relation between subjective perceptions of sleep and objectively measured sleep duration.

Therapeutic approaches target different predisposing, precipitating, and perpetuating factors thought to contribute to chronic insomnia. Predisposing factors include genetic susceptibility, heightened stress reactivity, and personality traits. Precipitating factors can be an illness, pain, acute stress, or a major life event. Perpetuating factors include poor sleep hygiene, maladaptive sleep behaviors, anxiety with excessive worry and monitoring of sleep, etc. Cognitive behavioral therapy (CBT-I), which targets the behavioral, cognitive, and physiological processes that maintain the disorder, is the recommended first-line therapy, and involves stimulus control, sleep restriction, relaxation training, cognitive restructuring, and sleep hygiene education. CBT-I exhibits robust efficacy in improving sleep quality, but its widespread implementation is limited by a shortage of trained providers and logistical barriers ^6^. Pharmacological therapies, including melatonin, hypnotics, and sedating antidepressants, are commonly prescribed for the management of insomnia and co-occurring depression. Although most clinical guidelines recommend these agents for short-term use only (typically ≤ 4 weeks), many patients remain on medication for substantially longer because of the limited availability of effective long-term alternatives. Prolonged use is associated with tolerance, drug dependence, cognitive and psychiatric adverse effects, and disruption of normal sleep architecture ^7^. Consequently, many patients continue to report dissatisfaction with their sleep ^8^, underscoring the need for safe, effective, and accessible non-pharmacological treatment options.

Transcranial electrical stimulation (tES) is increasingly being investigated for sleep modulation across both healthy and clinical populations due to its portability, favorable safety profile and ability to directly target sleep-related neural circuits and oscillations. tES is a form of non-invasive brain stimulation technique that can deliver low-intensity electrical currents (e.g. 1 to 2 mA) via scalp electrodes to modulate neuronal excitability changes in targeted cortical regions ^9^. Three different forms of tES, transcranial direct current stimulation (tDCS) ^10-12^, slow-oscillatory (so)-tDCS (~ 0.5–1 Hz) ^13-15^, and transcranial alternating current stimulation (tACS) ^16-19^, have been investigated for sleep promotion. in both healthy and clinical populations. Across studies, tES most commonly targeted frontal cortical regions (EEG sites: Fp1, Fp2, F3, F4) aiming to entrain slow-wave activity during non–rapid eye movement (NREM) deep sleep, grounded in the hypothesis that frontal lobe neurons exert “top-down” control over cortico-thalamic feedback loops that are essential for sleep-wake regulation ^11^. Several studies have reported increases in deep sleep duration, improvements in subjective sleep quality, and reductions in comorbid mood symptoms ^12,16,18,19^. However, findings remain heterogeneous. For example, bifrontal tDCS was reported to reduce total sleep time ^11^, whereas so-tDCS altered sleep-stage architecture without improving overall sleep duration ^14^ and was associated with reduced slow-wave sleep accompanied by impaired visuospatial memory consolidation ^15^. Similarly, one study demonstrated very low-frequency (0.75 and 1.0 Hz) sinusoidal tACS failed to entrain endogenous sleep oscillations ^20^ and another reported that neither 0.5 Hz nor 100 Hz tACS improved sleep quality over sham stimulation ^21^ .

In recent years, transcranial pulsed current stimulation (tPCS) has emerged as a novel form of tES. tPCS delivers low intensity pulsed currents (typically ≤ 2 mA average; 1–500 Hz) to modulate neuronal activity in targeted brain regions ^22-24^. Relative to tDCS, tPCS produces stronger neurophysiological effects and may recruit a broader range of sensory, motor, and nociceptive fibers at intensities as low as 0.3 mA ^22^. Compared to tACS, tPCS has a rapid onset of each pulse that creates a larger instantaneous change in the transmembrane electric field (*dE/dt*), which may be more effective at altering neuronal firing probability than the slowly varying sinusoidal waveform of tACS. Rather than aiming to entrain an endogenous rhythm, tPCS appears to act as a series of brief perturbations that may perturb network activity, making it less dependent on precise phase alignment with ongoing oscillations. Computational head-modeling of indicates that tPCS applied currents can reach deep subcortical structures, including the thalamus, hypothalamus, and brainstem ^25^, regions central to sleep–wake regulation. Mechanistically, tPCS may produce polarity-dependent modulation of cortical and corticospinal excitability^23^. Preclinical studies show that high-frequency (100-500Hz) pulsed electrical stimulation enhances GABAergic signaling through reduced GABA reuptake^26^ and interneuron activation^27^ while it suppresses neuronal firing, potentially through partial axonal conduction block ^28,29^.

The ability of higher frequency tPCS to enhance inhibitory control and suppress excessive excitatory activity may be particularly relevant for the treatment of insomnia, a disorder increasingly recognized as being driven by disrupted excitatory–inhibitory (E/I) balance and persistent hyperactivity in key arousal networks ^30^. In particular, wake-promoting neuronal populations within the ascending reticular activating system (ARAS), the principal brainstem arousal network, are thought to remain pathologically overactive because of insufficient GABAergic inhibition from the ventrolateral preoptic area (VLPO), which normally suppresses ARAS activity during sleep initiation and maintenance ^31^. Although these core sleep–wake nuclei are anatomically inaccessible to tES, the cerebellum represents an attractive target due to its superficial location and extensive reciprocal connections with the thalamus, hypothalamus, brainstem reticular formation and cerebral cortex involved in sleep regulation ^32,33^. Additionally, electrophysiological and neuroimaging studies demonstrate state-dependent modulation of cerebellar neuronal activity across the sleep–wake cycle^32^, while functional neuroimaging shows region-specific activation across cerebellar lobules during both NREM and REM sleep ^34^. Consistent with these observations, sleep fragmentation and REM sleep abnormalities are common in patients with cerebellar lesions or degenerative cerebellar ataxias, supporting a causal role for the cerebellum in sleep regulation^35^ .

Accordingly, we hypothesized that adjunctive bilateral cerebellar 400-Hz tPCS would increase objective actigraphy-derived total sleep time (TST), compared with sham tPCS in individuals with chronic insomnia. Actigraphy-derived TST was selected as the primary endpoint as objective sleep duration is more likely to capture the direct physiological effects of a brain stimulation intervention, such as cerebellar tPCS, on sleep regulation. We further hypothesized and prespecified as the key secondary endpoint that - relative to sham stimulation - adjunctive cerebellar 400-Hz tPCS would improve the overall patient-reported sleep quality, assessed using the Pittsburgh Sleep Quality Index (PSQI) global score.

## Results

**Figure 1:**
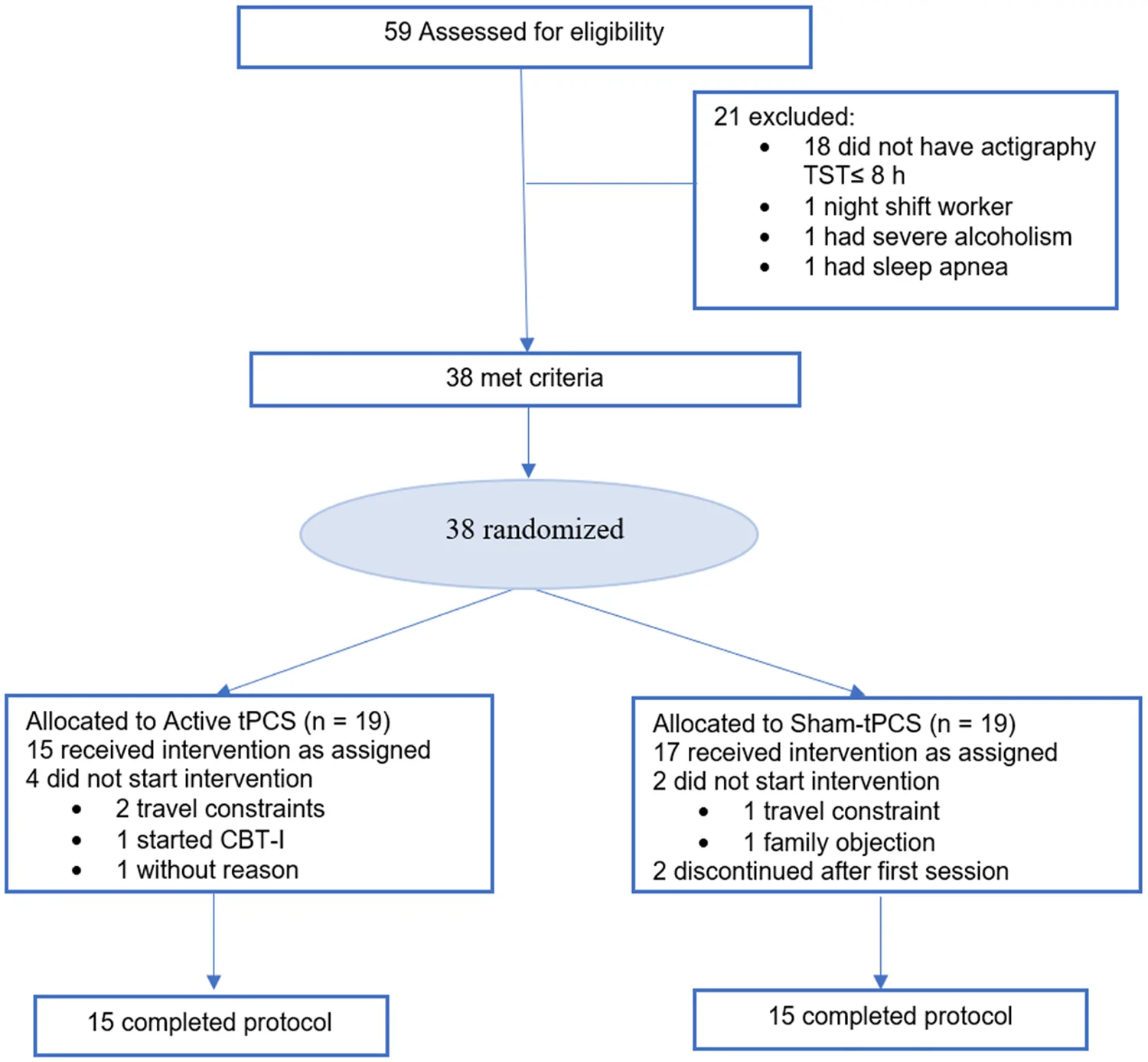
CONSORT flow diagram of the participants in the trial

A total of 59 individuals were screened for inclusion criteria eligibility and 21 were excluded. Participant flow is illustrated in the CONSORT diagram (Fig. 1). The remaining 38 participants were randomized to receive either active-tPCS or sham tPCS. During the trial, 6 participants (4 from active-tPCS and 2 from sham-tPCS group) withdrew (see Fig. 1) Consequently, 30 participants (9 males, 21 females; mean age 43 ± 12 years, range 24–70 years) completed the study, yielding a retention rate of 93.8% (30/32). Participant flow is illustrated in the CONSORT diagram (Fig. 1)

### Estimation of induced electric-field by 400Hz tPCS

**Figure 2:**
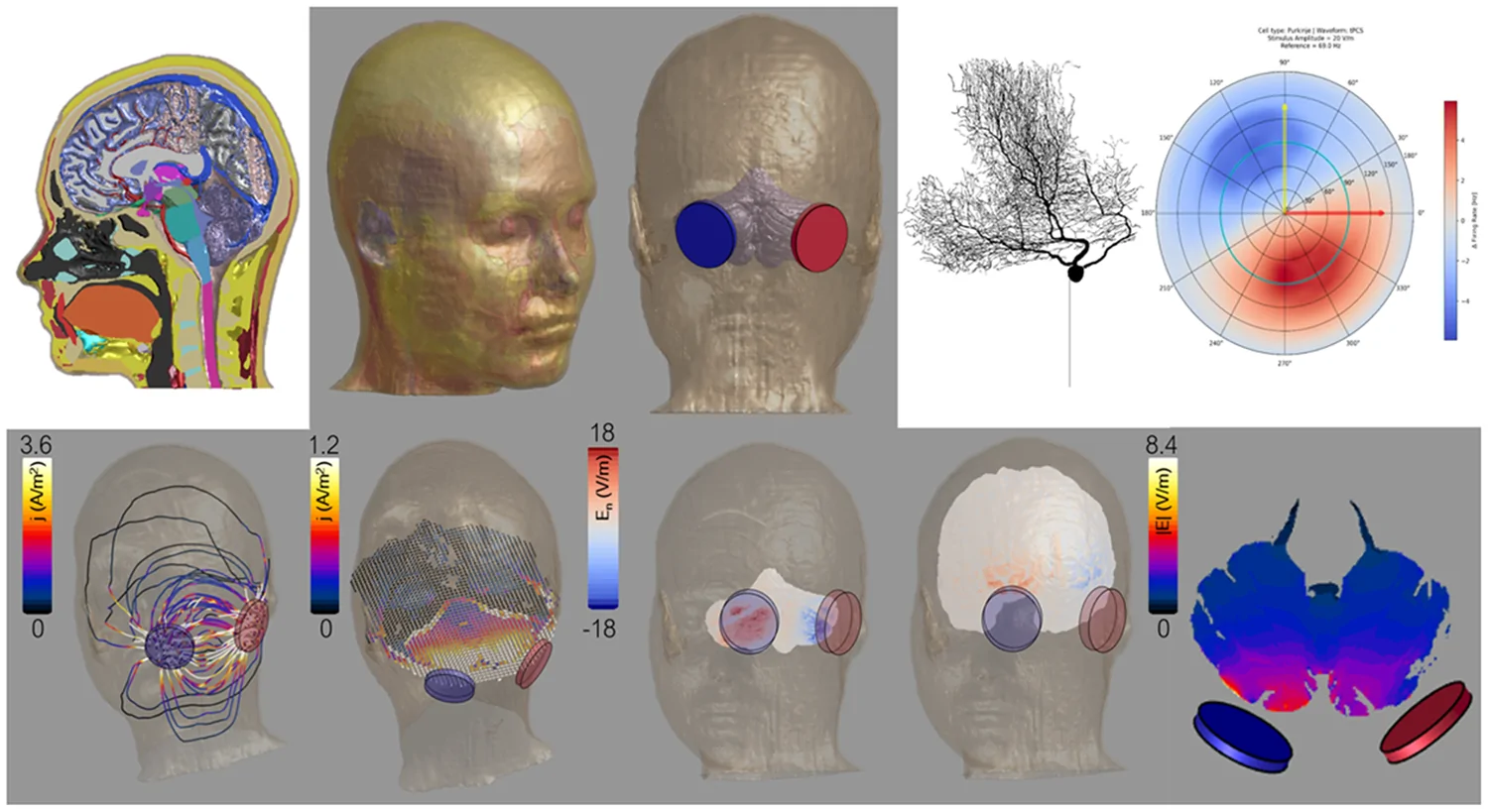
Estimation of induced electric-field by 400Hz tPCS Computational modeling of tPCS exposure; (top, left to right) cross section and 3D view of the MIDA anatomical head model; electrode placement on the model; morphology of the Purkinje cell model; dependence of modelled Purkinje cell firing rate change on relative field orientation at the predicted cerebellar tPCS exposure conditions; (bottom, left to right) streamline and vector field view of current flow colored by current density at pulse peak; normal E-field component surface view of the cerebellum and cerebrum; E-field magnitude cross-section view through the cerebellum.

Figure 2 shows current flow (colored by local current density distribution) together with cerebellar and brain surface maps of the normal electric field component, the principal exposure metric governing Purkinje cell firing modulation. Peak electric field magnitudes are shown at the temporal maximum of the stimulation pulse. Despite the highly folded cerebellar anatomy, distinct anodal and cathodal electric field distributions were observed beneath the respective electrodes. The highest induced cerebellar electric field magnitude occurred within the cerebellar cortex immediately beneath the electrodes and was estimated to alter the spontaneous firing rate of Purkinje cells by up to ± 5 Hz from a baseline rate of 69 Hz seen from the Purkinje cell model, depending on field orientation.

### Safety of tPCS

No serious adverse events occurred in either the active-tPCS or sham group during the study. The most reported adverse events were mild skin symptoms and mild headache. Their incidence did not differ significantly between groups (26.7% vs. 6.7%; RD = 20.0%, 95% CI − 5.7% to 45.7%; P = 0.330). Table 1 presents the between-group comparisons of SAEs. eTable 2a and eTable 2b in Supplement 3 presents the complete list of safety adverse event across all participants.

### Baseline comparison of demographic and clinical characteristics

Baseline demographic and clinical characteristics are presented in Table 2. Compared with the sham group, participants randomized to active tPCS were older (48.6 ± 10.7 vs. 37.7 ± 11.6 years; SMD = −1.02; 95% CI: −1.78, −0.25). No significant between-group differences were observed in sex, body mass index, educational attainment, or concomitant medication use. Relative to the sham group, the active-tPCS group exhibited shorter actigraphy-derived TST, shorter total time in bed, fewer nocturnal awakenings, and less WASO (SMDs, 1.21 to1.45). The active-tPCS group also had poorer perceived sleep quality and sleep efficiency (PSQI subjective sleep quality, SMD = 0.86; PSQI habitual sleep efficiency, SMD = 1.98), but lower self-rated anxiety and depression (SAS, SMD = 1.69; SDS, SMD = 2.01) scores.

### Effect of tPCS on Primary and Secondary Efficacy Outcomes

Post-treatment, between-group comparisons were performed using rank ANCOVA with corresponding baseline values as covariates because of the small sample size, non-normal data distribution, and baseline imbalances. Actigraphy-derived TST, the prespecified primary outcome, increased by 81.63 minutes (28.1%) in the active-tPCS group compared with 6.25 minutes (1.7%) in the sham group. Rank ANCOVA showed a significantly greater increase in TST with active tPCS after adjusting for baseline differences (adjusted mean difference = 34.25 minutes; 95% CI, 1.60–66.89; *P* = 0.022; partial η^2^ = 0.180). No otheractigraphy-derived secondary sleep outcomes remained statistically significant after Bonferroni correction.

Mean PSQI global score, the pre-specified key secondary outcome, decreased by 4.87 points (28.0%) in the active-tPCS group compared with 0.13 points (0.8%) in the sham group. Rank ANCOVA revealed a significantly greater improvement in the active-tPCS group (adjusted mean difference = − 4.39; 95% CI, − 6.47 to − 2.30; *P* < 0.001; partial η^2^ = 0.451). After Bonferroni correction, significant improvements remained for the PSQI subjective sleep quality and PSQI sleep duration subscales (adjusted mean differences = − 1.54 and − 1.49, respectively; both *P* < 0.001), favoring the active-tPCS group. No other PSQI component scores remained statistically significant after Bonferroni correction.

**Figure 3:**
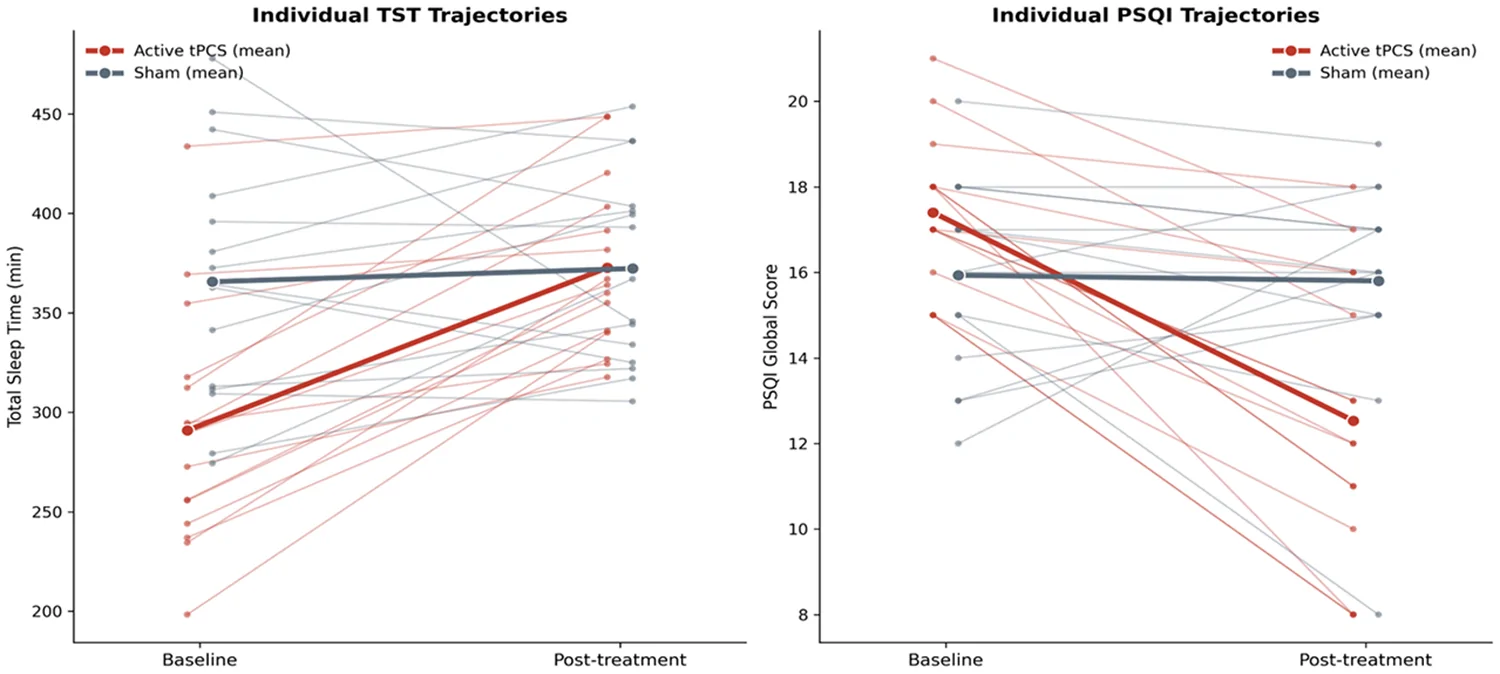
Individual subject trajectories from baseline to post-treatment change in actigraphy-derived TST and PSQI global score Individual trajectories are shown for actigraphy-derived total sleep time (TST; left) and Pittsburgh Sleep Quality Index (PSQI) global score (right) in the active tPCS and sham groups. Thin lines represent individual participants, and thick lines represent group means.

Figure 3 illustrates individual and mean trajectories for actigraphy-derived TST and PSQI global score. From baseline to post-treatment, the active-tPCS group demonstrated an upward shift in mean TST and a downward shift in mean PSQI global score, indicating longer sleep duration and improved overall sleep quality, respectively. In contrast, the sham group exhibited minimal change in either outcome, with relatively flat mean trajectories over time. Despite inter-individual variability, most participants in the active-tPCS group showed improvements consistent with the overall treatment effect.

Exploratory psychological outcomes were assessed using the SAS and SDS. Compared with the sham group, the active-tPCS group demonstrated greater reductions in both anxiety and depressive symptoms. Mean SAS scores decreased by 7.42 points (15.5%) in the active-tPCS group versus 0.67 points (1.0%) in the sham group. Rank ANCOVA revealed a significantly greater reduction in the active-tPCS after adjusting for baseline differences (adjusted mean difference = − 11.85; 95% CI, − 17.58 to − 6.12; P < 0.001; partial η^2^ = 0.409). Similarly, mean SDS scores decreased by 7.2 points (15.0%) in the active-tPCS group compared with 2.1 points (3.1%) in the sham group. Rank ANCOVA revealed a significantly greater reduction in the active-tPCS after adjusting for baseline differences (adjusted mean difference = − 12.13; 95% CI, − 17.24 to − 7.03; P < 0.001; partial η^2^ = 0.408). Post-treatment results for all outcomes are presented in Table 3.

### Sensitivity Analysis of Additional Baseline Covariates

Given the baseline imbalances between groups generated by the 1:1 randomization in the small sample size, an exploratory post hoc sensitivity analysis evaluated the impact of additional baseline variables as covariates in the rank ANCOVA models. Partial F-tests showed that none of the candidate variables explained significant additional variance in post-treatment TST or PSQI global score beyond treatment group and the corresponding baseline outcome (See Table 4).

## Discussion

Chronic insomnia is associated with substantial morbidity and increases long-term health risks ^3-5^. This single-centre, double-blind, sham-controlled randomized pilot trial evaluated the safety and efficacy of adjunctive high-frequency (400 Hz) cerebellar tPCS in individuals with chronic insomnia receiving stable pharmacotherapy.

Throughout the study, no serious adverse events occurred in either group, indicating that high-frequency cerebellar tPCS was well tolerated. Mild adverse events, including transient skin erythema and headache, were more frequent in the active tPCS group (4/15 vs 1/15; RD: 20.0% [− 5.7%, 45.7%]; P = 0.330), but overall adverse event incidence did not differ significantly between groups. These findings support the safety of cerebellar tPCS in adults with chronic insomnia.

Post-treatment, in the between-group rank ANCOVA adjusted for baseline actigraphy-derived TST to account for baseline between-group differences, the active-tPCS group demonstrated a significantly greater increase in TST compared to the sham group, which corresponded to an adjusted mean rank difference of 34.25 minutes. No other actigraphy-derived sleep parameter remained significant after Bonferroni correction. These findings suggest that 400 Hz cerebellar tPCS, when added to stable pharmacotherapy, may increase objective sleep duration in individuals with chronic insomnia.

The active-tPCS group also exhibited greater improvement in the pre-specified key secondary endpoint of overall subjective sleep quality compared to the sham group, with the between-group baseline-adjusted rank ANCOVA demonstrating significant between-group difference in PSQI global score at post treatment. This effect was mainly attributable to significant changes in the PSQI subcomponent scores of subjective sleep quality (P < 0.001, ES = 0.510) and sleep duration (P < 0.001, ES = 0.589), aligning with results of actigraphy. In addition, participants receiving active tPCS experienced significantly greater reductions in self-reported anxiety (SAS: P < 0.001, ES = 0.409) and depressive symptoms (SDS: P < 0.001, ES = 0.408) compared with those receiving sham stimulation.

Our results on the tPCS-induced improvement in subjective sleep measures are consistent with previous tES studies in individuals with sleep disturbances ^12,17,18^. However, the previous tES studies that primarily stimulated the frontal cortex generally reported limited or no effects on objective sleep measures ^10,14^. In contrast, cerebellar 400-Hz tPCS produced a significant increase in objective total sleep time alongside marked improvement in participants’ subjective perception of overall sleep quality. These results suggest that high-frequency cerebellar tPCS engages sleep–wake regulatory circuitry distinct from those modulated by frontal tES approaches, potentially reflecting differences in the target being the cerebellum, tPCS-specific stimulation characteristics and mechanisms of action. In any case, the concordance between objective and subjective sleep outcomes observed in the present study is clinically meaningful as these two dimensions of sleep are typically only weakly correlated ^36^ and their dissociation may contribute to sleep-state misperception, a characteristic feature of chronic insomnia associated with adverse health outcomes ^37^ .

Although the mechanisms by which high-frequency tPCS increased sleep duration remain incompletely understood, our electric-field modelling demonstrates substantial effects on Purkinje cells, which respond robustly to high-frequency stimulation ^38^. As the sole output neurons of the cerebellar cortex, Purkinje cells exert powerful inhibitory control over the deep cerebellar nuclei (DCN), the principal output structures of the cerebellum. The DCN project extensively to thalamic and brainstem regions, including components of the ARAS involved in sleep–wake regulation ^32,33^. Consequently, cerebellar 400-Hz tPCS may have influenced large-scale sleep–wake networks by modulating Purkinje cell activity and, in turn, cerebellar output to these downstream pathways.

Supporting this hypothesis, spontaneous Purkinje cell firing decreases during non-REM sleep in freely behaving cats ^39^, whereas experimentally increasing Purkinje cell activity suppresses DCN output and promotes arousal during transitions from non-REM sleep to wakefulness in mice ^40^. Together, these findings suggest that 400-Hz tPCS may have prolonged total sleep time by transiently suppressing Purkinje cell activity ^28,29^, thereby disinhibiting the DCN and enhancing downstream thalamic and brainstem pathways involved in sleep–wake regulation. Future studies incorporating polysomnography and neurophysiological measures will be required to delineate the specific circuit-level mechanisms underlying these effects.

Although exploratory, the observed reductions in anxiety and depressive symptoms following cerebellar 400-Hz tPCS are noteworthy given the high prevalence of comorbid affective symptoms among individuals with chronic insomnia ^41^. Extensive anatomical, neurophysiological, and imaging evidence reveals that activity in cerebellar hemispheres, particularly the posterior lobules and dentate nuclei, causally modulate contralateral prefrontal cortical activity^42^. Additionally, lateralized prefrontal cortical activity has been linked to mood regulation, with increased left prefrontal activity associated with antidepressant effects^43^. Accordingly, polarity-dependent modulation of Purkinje cell activity may have altered dentate output and, in turn, contralateral prefrontal cortical excitability, contributing to the mood improvements observed in this study. Nevertheless, whether lateralized cerebello–prefrontal network modulation contributes to the mood effects remains uncertain.

This study has several limitations. First, despite randomization, baseline imbalances were observed between groups in age, several actigraphy-derived sleep parameters, PSQI components, and anxiety and depression scores, with the active-tPCS group generally exhibiting greater baseline severity. To minimize potential confounding, between-group comparisons were performed using rank ANCOVA with adjustment for baseline values, and sensitivity analyses confirmed that the treatment effect on the primary outcome (total sleep time) and the key secondary outcome (PSQI global score) remained robust after accounting for potential confounding covariates. The robustness of our findings is supported by sensitivity analyses, which showed that none of the candidate baseline covariates significantly influenced the between-group differences in the primary outcome of actigraphy-derived TST or they key secondary outcome of PSQI global score (See Table 4), suggesting that observed effects were unlikely attributable to the baseline imbalances.

Second, participants remained on stable pharmacotherapy throughout the study because of the exploratory nature of high frequency tPCS and the need to maintain clinical stability in individuals who relied on these medications for daily functioning. Although concomitant sedative medications may have influenced objective sleep measures and introduced confounders, medication use was comparable between groups at baseline, reducing the likelihood that the observed treatment effects were attributable to differences in pharmacotherapy.

Third, although participant blinding was not formally assessed using a blinding questionnaire, the risk of unblinding was low because stimulation was delivered at low intensity, adverse events were mild, expected, and comparable between groups, and participants had no opportunity to interact during the study. In addition, stimulation technicians and all study personnel interacting with participants remained blinded to group allocation.

Fourth, owing to recruitment constraints, this pilot trial enrolled a relatively small sample, limiting statistical power and generalizability. Accordingly, these findings should be considered preliminary and require confirmation in larger, adequately powered randomized controlled trials.

Finally, polysomnography was not performed due to resource constraints, precluding assessment of sleep architecture and preventing determination of whether the observed increase in total sleep time was attributable to specific sleep stages, such as slow-wave sleep.

In conclusion, this pilot double-blind, randomized, sham-controlled trial demonstrated that adjunct cerebellar 400-Hz tPCS was safe and significantly improved both objective actigraphy-derived total sleep time and subjective sleep quality in individuals with chronic insomnia as compared with sham stimulation. Improvements seem clinically meaningful, as objective and subjective sleep measures often correlate only weakly, and their discordance contributes to sleep misperception in individuals with insomnia. Exploratory analyses further suggested potential benefits for comorbid anxiety and depressive symptoms. Collectively, these findings identify the cerebellum as a promising therapeutic target for chronic insomnia and support further evaluation of high-frequency cerebellar tPCS as a non-pharmacological adjunct to standard care in larger, adequately powered trials incorporating longer follow-up, and assessment of potential medication-sparing effects.

## Methods

### Study Design

A two-week, single-center, parallel-group, double-blind, randomized, sham-controlled clinical trial was conducted at Chongqing Western Hospital, Sichuan Province, China. Eligible participants who met the criteria and provided written informed consent were randomized 1:1 to receive active 400Hz tPCS or sham stimulation. The study was approved by the institutional ethics committee, registered at the Chinese Clinical Trial Registry (ChiCTR2300072403), and conducted in accordance with the Declaration of Helsinki.

### Participants

Participants with chronic insomnia were recruited from the outpatient sleep clinic of Chongqing Western Hospital, Chongqing, China, between June 19, 2023, and May 30, 2025. Chronic insomnia was diagnosed according to established clinical criteria ^2^ as persistent difficulty initiating or maintaining sleep, or early morning awakening despite adequate opportunity for sleep, occurring ≥ 3 nights per week for ≥ 3 months and associated with clinically significant daytime impairment. Eligible participants underwent three consecutive nights of home wrist actigraphy (ActiGraph wGT3X-BT), and only those with an average whole-night actigraphy-derived total sleep time (TST) ≤ 480 minutes (≤ 8 hours) were randomized to active or sham tPCS. This TST threshold was selected because participants remained on stable medications, some of which had sedative effects that could reduce movement while awake and cause actigraphy to misclassify periods of quiet wakefulness as sleep, thereby overestimating TST.

Inclusion criteria were age 18–65 years, PSQI score 11–21, stable insomnia medication for ≥ 30 days. Individuals with mild to moderately severe anxiety and depressive symptoms (DSM-5 criteria) ^44^ were eligible. Women of childbearing potential required a negative pregnancy test. Exclusion criteria included major psychiatric disorders; history of seizures or craniotomy; implanted electronic or ferromagnetic devices; other primary sleep disorders (e.g., obstructive sleep apnea, periodic limb movement disorder, narcolepsy, parasomnia, or hypersomnia); active suicidal ideation; clinically significant medical or neurological illness; substance dependence; night-shift work; pregnancy or breastfeeding; recent non-invasive brain stimulation (e.g., rTMS or tDCS); concurrent psychological therapy (e.g., CBT-I); or participation in another interventional study.

### Sample size calculation

The original sample size was 130 participants (65 per group), calculated to provide 80% power at a two-sided α of 0.05 while allowing for 20% attrition. Recruitment constraints prevented attainment of the planned sample within the approved study period. Consequently, the study was completed as a pilot randomized controlled trial to inform a future fully powered trial, with a target of 30 evaluable participants (15 per group).

### Randomization and Blinding

A total of 38 eligible participants were randomized 1:1 to receive either active or sham tPCS using a computer-generated randomization sequence. Sequence generation and allocation were performed by an independent statistician who was not involved in participant enrolment, intervention delivery, or outcome assessment. Allocation concealment was maintained using sequentially numbered, sealed, opaque envelopes opened in order of enrolment. Active and sham devices were identical in appearance and stimulation was administered in separate treatment rooms with comparable layouts located in different areas of the hospital. Participants’ visits were scheduled at least two hours apart, and each participant attended all 12 sessions in the same treatment room to minimize contact and reduce the risk of accidental unblinding. All participants were tPCS-naïve and were informed that transient stimulation-related sensations, including mild skin tingling, itching, or headache, could occur during either active or sham stimulation to preserve blinding. Therapists administering the intervention and study personnel responsible for outcome assessments remained blinded to group allocation throughout the trial.

### Outcome Measures

The primary outcome was actigraphy-derived total sleep time (TST), calculated as the mean of three consecutive nights of whole-night recordings using wrist actigraphy (ActiGraph wGT3X-BT; ActiGraph LLC, Pensacola, FL, USA) worn on the non-dominant wrist. Actigraphy is a validated method for assessing sleep–wake patterns and demonstrates good agreement with polysomnography for estimating TST, particularly in naturalistic settings ^45^. Baseline measurements were obtained during the week preceding the first treatment session, and post-treatment TST was calculated as the mean of the final three nights of the intervention.

The key secondary outcome was the Chinese version of the Pittsburgh Sleep Quality Index (PSQI) ^46^ global score. Higher PSQI scores indicate poorer sleep quality (global score range, 0–21; scores > 5 indicate poor sleep quality). Additional secondary outcomes included actigraphy-derived sleep efficiency (SE), sleep latency (SL), total time in bed (TIB), wake after sleep onset (WASO), number of awakenings, average awakening duration, the seven PSQI component scores, and mood symptoms assessed using the Chinese versions of the Zung Self-Rating Depression Scale (SDS) ^47^ and Zung Self-Rating Anxiety Scale (SAS) ^48^. Higher SDS and SAS scores indicate greater depression and anxiety severity, respectively.

Baseline assessments were performed at Week 0. Post-treatment actigraphy outcomes were derived from the final three intervention days, whereas the PSQI, SDS, and SAS were completed within one week after the final treatment session under the supervision of trained psychologists blinded to treatment allocation.

### Intervention

Stimulation was delivered using a transcranial pulsed current stimulator (YQ-D1111; YIQI Biotechnology Ltd., China). Participants underwent 12 daily treatment sessions of 30 minutes each over two consecutive weeks (Monday–Saturday). For each participant, all sessions were applied at a consistent time. 400-Hz tPCS was delivered via two saline-soaked circular silver–fiber cotton electrodes (12.56 cm^2^) positioned bilaterally over the cerebellar hemispheres (1 cm inferior and 4 cm lateral to the inion), with the cathode over the left and the anode over the right hemisphere. The active group received continuous stimulation at 0.6 mA, whereas the sham group received a 10-s ramp-up followed by a 5-s ramp-down with no subsequent stimulation. Participants were permitted to continue their existing pharmacotherapy. Changes in medications during the study were not permitted.

### Computational Exposure Assessment

Electromagnetic field simulations were performed using Sim4Life V8.2 (ZMT, Zurich, Switzerland) and the high-resolution MIDA anatomical model ^49^ to estimate cerebellar and whole-brain exposure to tPCS. The clinical electrode montage was reproduced, with tissue conductivities assigned according to the IT’IS Tissue Properties Database v4.2. To estimate neuronal effects, a morphologically and electrophysiologically detailed Purkinje cell model from ModelDB ^50^was coupled to the simulated extracellular electric fields. Changes in spontaneous firing rate (baseline for the Purkinje cell model: 69 Hz) were evaluated as a function of electric field strength and orientation.

### Safety monitoring

Adverse events were systematically monitored in both groups. After each session, participants were assessed for treatment-related adverse events, including dizziness, headache, vomiting, seizures, scalp burns, skin redness or itching, scalp or neck pain, and other symptoms. All adverse events were documented, and any serious adverse events were reported in accordance with institutional procedures using standardized hospital reporting forms.

### Statistical Methods

All analyses were performed using IBM SPSS Statistics (version 23.0; IBM Corp., Armonk, NY, USA) and R (version 4.2.2; R Foundation for Statistical Computing, Vienna, Austria) in the per-protocol population. Baseline balance between groups was assessed using standardized mean differences (SMD), calculated as the difference in means (or proportions) divided by the pooled standard deviation, given the reduced sensitivity of hypothesis testing to imbalance at small sample sizes. Variables with SMD > 0.8 were considered for inclusion as covariates in subsequent adjusted analyses. Normality was assessed using the Shapiro–Wilk and Kolmogorov–Smirnov tests (see Supplement 3, eTable 1). As several variables were non-normally distributed and baseline imbalances were present, post-treatment between-group comparisons were performed using rank analysis of covariance (rank ANCOVA), adjusting for baseline values. All tests were two-sided with a significance level of *P* < 0.05. Bonferroni correction was applied to the six secondary actigraphy-derived sleep parameters and the PSQI global and seven component scores. Actigraphy-derived TST was prespecified as the primary outcome and was not adjusted for multiplicity. Between-group adverse events incidence was compared using chi-square.

Exploratory sensitivity analyses evaluated whether baseline imbalances (age, sleep parameters, PSQI components, SAS, and SDS) had confounding effect on primary outcome of post-treatment TST or key secondary outcome of PSQI global score. Hierarchical rank-based linear regression models were used to test whether any variable showing significant between-group differences at baseline explained additional variance in post-treatment TST (Post-TST) and PSQI global scores beyond treatment group and the corresponding rank-transformed baseline outcome (baseline TST for Post-TST; baseline PSQI global score for Post-PSQI). The incremental contribution was evaluated using the change in explained variance (ΔR^2^) and the associated F-change statistic. A significant ΔR^2^ (p < 0.05) indicated that the covariate accounted for unique variability in post-treatment outcomes; partial eta squared (ηp^2^) quantified the magnitude of this unique effect.

## Supplementary Material

This is a list of supplementary files associated with this preprint. Click to download.

- Supplement1Trialprotocol.pdf
- Supplement2CONSORT2025checklistCerebellarHighFrequencyTranscranialPulsedCurrentStimulationIncreasesTotalSleepTimeinChronicInsomniaApilotst
- Supplement3eTABLES.pdf

## Figures and Tables

**Table 1:** Between-group comparison of adverse events

Item	tPCS group [n (%)]	Sham group [n (%)]	RD (95% CI)	P (Fisher)
Week 1 & 2 – Skin symptoms	3 (20.0%)	0 (0.0%)	20.0% (− 0.2%, 40.2%)	0.224
Week 1 & 2 – Mild headache	2 (13.3%)	1 (6.7%)	6.7% (− 14.7%, 28.0%)	1.000
Any skin symptoms	3 (20.0%)	0 (0.0%)	20.0% (− 0.2%, 40.2%)	0.224
Any headache	2 (13.3%)	1 (6.7%)	6.7% (− 14.7%, 28.0%)	1.000
Overall adverse events	4 (26.7%)	1 (6.7%)	20.0% (− 5.7%, 45.7%)	0.330

Note. n = 15 per group. RD = risk difference (tPCS – Sham), reported with 95% Wald confidence interval; P-values from two-tailed Fisher’s exact test. * P < 0.05

Participants reporting a given symptom on more than one occasion (e.g., in both Week 1 and Week 2) were counted once (coded as 1) rather than by number of episodes. “Overall adverse events” reflects the number of unique participants reporting at least one adverse event (skin symptoms and/or headache) across the study period.

**Table 2:** Baseline demographic and clinical characteristics

	Group				
	Overall N = 30	Sham N = 15	Active-tPCS N = 15	SMD	95% CI
**Demographic & Anthropometrics**					
Sex, n (%)				0.45	−0.28,1.17
female	21 (70.00%)	12 (80.00%)	9 (60.00%)		
male	9 (30.00%)	3 (20.00%)	6 (40.00%)		
Educational level, n (%)				0.59	−0.14,1.32
Junior high school or below	10 (33.33%)	3 (20.00%)	7 (46.67%)		
Senior high school or above	20 (66.67%)	12 (80.00%)	8 (53.33%)		
Age, Mean ± SD	43.13 ± 12.28	37.67 ± 11.60	48.60 ± 10.68	−1.02	−1.78, −0.25
Height (cm), Mean ± SD	163.27 ± 8.30	161.87 ± 7.37	164.67 ± 9.17	−0.35	−1.07, 0.37
Weight (kg), Mean ± SD	55.84 ± 7.92	55.07 ± 7.45	56.62 ± 8.56	−0.20	−0.92, 0.52
BMI, Mean ± SD	20.93 ± 2.36	21.02 ± 2.53	20.84 ± 2.26	0.08	−0.64, 0.80
Actigraphy					
Actigraphy TST, mean ± SD	328.26 ± 71.28	365.62 ± 62.11	290.90 ± 60.62	1.26	0.48, 2.04
Actigraphy SL, median (Q1, Q3)	5.50 (3.33, 7.33)	4.17 (2.67, 7.67)	6.00 (4.00, 7.33)	0.04	−0.67, 0.76
Actigraphy SE, mean ± SD	86.34 ± 6.39	84.28 ± 7.58	88.40 ± 4.25	−0.69	−1.43, 0.04
Actigraphy TIB, mean ± SD	383.06 ± 92.74	436.55 ± 81.10	329.58 ± 71.48	1.45	0.64, 2.25
Actigraphy WASO, median (Q1, Q3)	33.67 (26.67, 70.33)	64.67 (30.00, 89.00)	27.00 (22.67, 36.00)	1.12	0.35,1.89
Actigraphy No. of awakening, median (Q1, Q3)	16.08 (10.67, 20.60)	19.00 (15.33, 28.14)	11.33 (9.67, 17.67)	1.21	0.43,1.99
Actigraphy Avg. awakening (minutes), median (Q1, Q3)	2.60 (2.11, 3.25)	2.64 (2.15, 3.84)	2.53 (2.10, 3.24)	0.37	−0.35,1.09
PSQI					
PSQI global, median (Q1, Q3)	17.00 (15.00, 18.00)	16.00 (14.00, 18.00)	17.00 (16.00, 18.00)	−0.75	−1.49, −0.01
Subjective sleep quality, n (%)				0.86	0.11,1.61
2 points (fairly bad)	7 (23.3%)	6 (40.0%)	1 (6.7%)		
3 points (very bad)	23 (76.7%)	9 (60.0%)	14 (93.3%)		
Sleep latency, n (%)				0.63	−0.10,1.37
2 points (moderate difficulty)	8 (26.7%)	6 (40.0%)	2 (13.3%)		
3 points (severe difficulty falling asleep)	22 (73.3%)	9 (60.0%)	13 (86.7%)		
Sleep duration, n (%)				0.58	−0.15,1.31
1 point (6–7 hours)	2 (6.7%)	0 (0.0%)	2 (13.3%)		
2 points (5–6 hours)	12 (40.0%)	7 (46.7%)	5 (33.3%)		
3 points (< 5 hours)	16 (53.3%)	8 (53.3%)	8 (53.3%)		
Habitual sleep efficiency, n (%)				1.98	1.11, 2.85
0 points (≥ 85% sleep efficiency)	7 (23.3%)	7 (46.7%)	0 (0.0%)		
1 point (75–84%)	3 (10.0%)	1 (6.7%)	2 (13.3%)		
2 points (65–74%)	7 (23.3%)	5 (33.3%)	2 (13.3%)		
3 points (< 65%)	13 (43.3%)	2 (13.3%)	11 (73.3%)		
Sleep disturbances, n (%)				0.57	−0.16, 1.30
1 point (mild sleep disturbances)	10 (33.3%)	4 (26.7%)	6 (40.0%)		
2 points (moderate sleep disturbances)	15 (50.0%)	7 (46.7%)	8 (53.3%)		
3 points (severe sleep disturbances)	5 (16.7%)	4 (26.7%)	1 (6.7%)		
Use of sleep medication, n (%)				0.40	−0.32, 1.12
2 points (once or twice a week)	4 (13.3%)	3 (20.0%)	1 (6.7%)		
3 points (three or more times a week)	26 (86.7%)	12 (80.0%)	14 (93.3%)		
Daytime dysfunction, n (%)				0.94	0.19,1.70
0 points (no daytime dysfunction)	1 (3.3%)	1 (6.7%)	0 (0.0%)		
1 point (mild daytime dysfunction)	2 (6.7%)	0 (0.0%)	2 (13.3%)		
2 points (moderate daytime dysfunction)	19 (63.3%)	8 (53.3%)	11 (73.3%)		
3 points (severe daytime dysfunction)	8 (26.7%)	6 (40.0%)	2 (13.3%)		
Psychological Outcomes					
SAS, median (Q1, Q3)	53.75 (48.00, 62.50)	60.00 (55.00, 71.25)	48.00 (40.00, 53.75)	1.69	0.86, 2.53
SDS, median (Q1, Q3)	55.63 (47.25, 67.25)	67.25 (57.50, 82.50)	47.25 (41.25, 55.00)	2.01	1.13, 2.89
Concomitant Medications					
Benzodiazepine Use, n (%)	17 (56.7%)	8 (53.3%)	9 (60.0%)	0.13	−0.58, 0.85
Non-benzodiazepine hypnotics (Z-drugs)	8 (26.7%)	2 (13.3%)	6 (40.0%)	0.63	−0.10, 1.37
Sedating SSRIs/SARI/TCA	6 (20.0%)	5 (33.3%)	1(6.7%)	0.71	−0.03, 1.44
Minimally sedating SSRIs/SARI/TCA	6 (20.0%)	5 (33.3%)	1 (6.7%)	0.71	−0.03, 1.44
Sedating antipsychotics	11 (36.7%)	5 (33.3%)	6 (40.0%)	0.14	−0.58, 0.86
Non-sedating antipsychotics	2 (6.7%)	1 (6.7%)	1 (6.7%)	0.00	−0.72, 0.72

Abbreviations:

BMI = body mass index; SD = standard deviation; TST = total sleep time; SL= sleep latency; SE = sleep efficiency; TIB = time in bed; WASO = wake after sleep onset; SAS- Zung Self-Rating Anxiety Scale; SDS = Zung Self-Rating Depression Scale; PSQI-Pittsburg Sleep Quality Index. SSRI = selective serotonin reuptake inhibitor; SARI = serotonin antagonist and reuptake inhibitor; TCA = tricyclic antidepressant; CI = Confidence Interval.

Threshold: ∣SMD∣ > 0.1 suggests imbalance, > 0.2 small, > 0.5 moderate, > 0.8 large.

**Table 3:** Post-treatment Outcomes analyzed using rank ANCOVA

Outcome	Mean ± SD		Adjusted Mean Rank^a^ ± SD		Adjusted Mean Rank Diff Btw Grp (95% CI)	P value^b^	Effect Size^c^
	Sham	Active-tpcs	Sham	Active-tpcs			
**Actigraphy**							
Actigraphy TST, Mean (SD)	372.25 ± 48.60	372.63 ± 42.51	355.32 ± 10.42	389.57 ± 10.42	34.25 (1.60, 66.89)	0.022	0.180
Actigraphy SL, Mean (SD)	4.79 ± 3.48	4.98 ± 1.61	4.78 ± 0.70	4.99 ± 0.70	0.21 (− 1.81, 2.24)	0.995	0.000
Actigraphy SE, Mean (SD)	82.95 ± 6.43	88.29 ± 3.25	84.08 ± 1.03	87.16 ± 1.03	3.08 (0.00, 6.16)	0.030	0.163
Actigraphy TIB, Mean (SD)	449.37 ± 49.25	422.73 ± 50.82	424.85 ± 10.55	447.26 ± 10.55	22.42 (− 11.22, 56.06)	0.432	0.023
Actigraphy WASO, Mean (SD)	72.34 ± 29.31	45.12 ± 16.77	63.54 ± 4.96	53.92 ± 4.96	−9.62 (− 24.97, 5.72)	0.083	0.107
Actigraphy No. of Awakening, Mean (SD)	22.25 ± 6.46	18.42 ± 6.03	20.37 ± 1.53	20.29 ± 1.53	−0.08 (− 4.86, 4.70)	0.520	0.016
Actigraphy Avg. Awakening (min), Mean (SD)	3.26 ± 1.16	2.54 ± 0.63	3.13 ± 0.15	2.67 ± 0.15	−0.46 (− 0.91, − 0.02)	0.017	0.194
PSQI Outcomes							
PSQI Global, Mean (SD)	15.80 ± 2.62	12.53 ± 3.31	16.36 ± 0.70	11.97 ± 0.70	−4.39 (− 6.47, − 2.30)	< 0.001	0.451
Subjective Sleep Quality, Mean (SD)	2.60 ± 0.83	1.27 ± 0.88	2.71 ± 0.23	1.16 ± 0.23	−1.54 (− 2.22, − 0.87)	< 0.001	0.510
Sleep Latency, Mean (SD)	2.60 ± 0.51	2.07 ± 0.88	2.69 ± 0.18	1.97 ± 0.18	−0.72 (− 1.24,−0.20)	0.016	0.195
Sleep Duration, Mean (SD)	2.73 ± 0.46	1.20 ± 0.86	2.71 ± 0.17	1.22 ± 0.17	−1.49 (− 1.99, − 0.99)	< 0.001	0.589
Habitual Sleep Efficiency, Mean (SD)	1.20 ± 1.21	1.53 ± 1.06	1.65 ± 0.29	1.08 ± 0.29	−0.57 (− 1.49, 0.36)	0.216	0.056
Sleep Disturbances, Mean (SD)	2.00 ± 0.85	1.13 ± 0.52	1.90 ± 0.15	1.23 ± 0.15	−0.68 (− 1.13, − 0.22)	0.007	0.244
Use of Sleep Medication, Mean (SD)	2.53 ± 0.83	2.67 ± 0.90	2.58 ± 0.22	2.62 ± 0.22	0.04 (− 0.61, 0.69)	0.422	0.024
PSQI Daytime Dysfunction, Mean (SD)	2.13 ± 0.92	1.33 ± 0.62	2.10 ± 0.20	1.37 ± 0.20	−0.73 (− 1.32, − 0.14)	0.021	0.183
Psychiatric Scales							
SAS, Mean (SD)	62.33 ± 8.97	40.58 ± 7.66	57.38 ± 1.76	45.54 ± 1.76	−11.85 (− 17.58, − 6.12)	< 0.001	0.409
SDS, Mean (SD)	69.08 ± 11.89	40.83 ± 6.38	61.02 ± 1.52	48.89 ± 1.52	−12.13 (− 17.24, − 7.03)	< 0.001	0.408

a Adjusted mean rank represents the baseline value adjusted average rank of the outcome derived from the Rank ANCOVA model.

b P-values were obtained using Rank ANCOVA model.

c Effect sizes represent partial η^2^. Partial n^2^ effect size: Small effect = ~ 0.01; Medium effect = ~ 0.06; Large effect = ≥ 0.14. Multiplicity adjustment (See Supplement 3, eTable 3)

**Table 4:** Incremental candidate covariates for Post-treatment TST and Post-treatment PSQI global score

Candidate Covariate	F(1,26) (Post-TST)	ΔR^2^ (Post-TST)	P-value (Post-TST)	Partial η^2^ (Post-TST)	F(1,26) (Post-PSQI Global)	ΔR^2^ (Post-PSQI Global)	P-value (Post-PSQI Global)	Partial η^2^ (Post-PSQI Global)
AGE	1.204	0.026	0.283	0.044	0.536	0.010	0.471	0.020
Baseline TST (actigraphy)	n.a	n.a.	n.a	n.a.	0.365	0.007	0.551	0.014
Baseline TIB	2.147	0.046	0.155	0.076	0.444	0.008	0.511	0.017
Baseline WASO	0.342	0.008	0.564	0.013	0.475	0.009	0.497	0.018
Baseline No. of Awakenings	0.242	0.006	0.627	0.009	0.035	0.001	0.853	0.001
PSQI Sleep Quality (baseline)	0.083	0.002	0.776	0.003	0.327	0.006	0.572	0.012
PSQI Sleep Efficiency (baseline)	0.411	0.009	0.527	0.016	0.123	0.002	0.729	0.005
Baseline SAS	0.000	0.000	0.983	0.000	0.422	0.008	0.522	0.016
Baseline SDS	0.849	0.019	0.365	0.032	2.161	0.038	0.154	0.077

Each candidate covariate (showing significant between-group differences at baseline) was added separately to the model, and its incremental contribution was evaluated using the change in explained variance (ΔR^2^) and the associated F-change statistic. A significant ΔR^2^ (P < 0.05) indicated that the covariate accounted for unique variability in post-treatment sleep outcomes beyond treatment allocation and baseline severity; partial eta squared (ηp^2^) quantified the magnitude of this unique effect.

## Data Availability

The data underlying this article are available in the Research Manager (ResMan) repository of Clinical Trial Management Public Platform, at http://www.medresman.org.cn/uc/projectsh/projectedit.aspx?proj=6304

## References

[1] Ohayon M. M. Epidemiological overview of sleep disorders in the general population. Sleep. Med. Res. 2, 1–9 (2011).

[2] Sateia M. J. International classification of sleep disorders-third edition. Chest 146, 1387–1394 (2014). 10.1378/chest.14-0970 25367475

[3] Baglioni C. Insomnia as a predictor of depression: a meta-analytic evaluation of longitudinal epidemiological studies. J. Affect. Disord. 135, 10–19 (2011). 10.1016/j.jad.2011.01.011 21300408

[4] Saint-Maurice P. F. Associations between actigraphy-measured sleep duration, continuity, and timing with mortality in the UK Biobank. Sleep 47 (2024). 10.1093/sleep/zsad312 PMC10925955 38066693

[5] Parthasarathy S. Persistent insomnia is associated with mortality risk. Am. J. Med. 128, 268–275e2 (2015). 10.1016/j.amjmed.2014.10.015 PMC4340773 25447616

[6] Koffel E., Bramoweth A. D. & Ulmer C. S. Increasing access to and utilization of cognitive behavioral therapy for insomnia (CBT-I): a narrative review. J. Gen. Intern. Med. 33, 955–962 (2018). 10.1007/s11606-018-4390-1 PMC5975165 29619651

[7] Johnson B. & Streltzer J. Risks associated with long-term benzodiazepine use. Am. Fam Physician. 88, 224–226 (2013). 23944724

[8] Krakow B., Ulibarri V. A. & Romero E. Persistent insomnia in chronic hypnotic users presenting to a sleep medical center. J. Nerv. Ment Dis. 198, 734–741 (2010). 10.1097/NMD.0b013e3181f4aca1 20921864

[9] Nitsche M. A. Transcranial direct current stimulation: state of the art 2008. Brain Stimul. 1, 206–223 (2008). 10.1016/j.brs.2008.06.004 20633386

[10] Frase L. Differential effects of bifrontal tDCS on arousal and sleep duration in insomnia patients and healthy controls. Brain Stimul. 12, 674–683 (2019). 10.1016/j.brs.2019.01.001 30639236

[11] Frase L. Modulation of total sleep time by transcranial direct current stimulation (tDCS). Neuropsychopharmacology 41, 2577–2586 (2016). 10.1038/npp.2016.65 PMC4987856 27143601

[12] Zhou Q. The effects of repeated transcranial direct current stimulation on sleep quality and depression symptoms in patients with major depression and insomnia. Sleep. Med. 70, 17–26 (2020). 10.1016/j.sleep.2020.02.003 32179428

[13] Marshall L., Helgadóttir H., Mölle M. & Born J. Boosting slow oscillations during sleep potentiates memory. Nature 444, 610–613 (2006). 10.1038/nature05278 17086200

[14] Saebipour M. R. Slow oscillating transcranial direct current stimulation during sleep has a sleep-stabilizing effect in chronic insomnia: a pilot study. J. Sleep. Res. 24, 518–525 (2015). 10.1111/jsr.12301 26014344

[15] Paßmann S. Boosting slow oscillatory activity using tDCS during early nocturnal slow wave sleep does not improve memory consolidation in healthy older adults. Brain Stimul. 9, 730–739 (2016). 10.1016/j.brs.2016.04.016 27247261

[16] Wang H. X. Effect of transcranial alternating current stimulation for the treatment of chronic insomnia: a randomized, double-blind, parallel-group, placebo-controlled clinical trial. Psychother. Psychosom. 89, 38–47 (2020). 10.1159/000504609 31846980

[17] Zhu X., Ren Y., Tan S. & Ma X. Efficacy of transcranial alternating current stimulation in treating chronic insomnia and the impact of age on its effectiveness: a multisite randomized, double-blind, parallel-group, placebo-controlled study. J. Psychiatr Res. 170, 253–261 (2024). 10.1016/j.jpsychires.2023.12.037 38176353

[18] Robinson C. S. H. The benefits of closed-loop transcranial alternating current stimulation on subjective sleep quality. Brain Sci. 8, 204 (2018). 10.3390/brainsci8120204 PMC6316321 30469495

[19] Hathaway E. Transcranial electrical stimulation targeting limbic cortex increases the duration of human deep sleep. Sleep. Med. 81, 350–357 (2021). 10.1016/j.sleep.2021.03.001 PMC8108560 33812203

[20] Lafon B. Low frequency transcranial electrical stimulation does not entrain sleep rhythms measured by human intracranial recordings. Nat. Commun. 8, 1199 (2017). 10.1038/s41467-017-01045-x PMC5662600 29084960

[21] Lee M., Hong J. K., Lee Y. & Yoon I. Y. Transcranial alternating current stimulation in subjects with insomnia symptoms: a randomized, double-blind and controlled study. J. Psychiatr Res. 186, 129–136 (2025). 10.1016/j.jpsychires.2025.04.020 40239389

[22] Jaberzadeh S. & Zoghi M. Exploring sensory, motor, and pain responses as potential side or therapeutic effects of sub-2 mA, 400 Hz transcranial pulsed current stimulation. PLoS One. 18, e0290137 (2023). 10.1371/journal.pone.0290137 PMC10718437 38091312

[23] Jaberzadeh S., Malekahmad M., Liu R. & Zoghi M. Effects of a single session 400 Hz transcranial pulsed current stimulation on corticospinal and corticocortical excitability and hand dexterity: a double-blind RCT. Psychophysiology 62 (2025). 10.1111/psyp.70112 PMC12284344 40696723

[24] Liu Z. Transcranial pulsed current stimulation and social functioning in children with autism: a randomized clinical trial. JAMA Netw. Open. 8, e255776 (2025). 10.1001/jamanetworkopen.2025.5776 PMC12013354 40257798

[25] Datta A., Dmochowski J. P., Guleyupoglu B., Bikson M. & Fregni F. Cranial electrotherapy stimulation and transcranial pulsed current stimulation: a computer based high-resolution modeling study. Neuroimage 65, 280–287 (2013). 10.1016/j.neuroimage.2012.09.062 23041337

[26] Li T., Qadri F. & Moser A. Neuronal electrical high frequency stimulation modulates presynaptic GABAergic physiology. Neurosci. Lett. 371, 117–121 (2004). 10.1016/j.neulet.2004.08.050 15519740

[27] Ye X. Activating interneurons in local inhibitory circuits by high-frequency stimulations at the efferent fibers of pyramidal neurons in rat hippocampal CA1 region. Brain Sci. 12, 1350 (2022). 10.3390/brainsci12101350 PMC9599906 36291284

[28] Feng Z. High frequency stimulation of afferent fibers generates asynchronous firing in the downstream neurons in hippocampus through partial block of axonal conduction. Brain Res. 1661, 67–78 (2017). 10.1016/j.brainres.2017.02.008 28213155

[29] Yuan Y., Feng Z., Yang G., Ye X. & Wang Z. Suppression of neuronal firing following antidromic high-frequency stimulations on the neuronal axons in rat hippocampal CA1 region. Front Neurosci 16 (2022). 10.3389/fnins.2022.881426 PMC9226389 35757541

[30] Arias-Carrión O. Redefining insomnia: from neural dysregulation to personalized therapeutics. Expert Rev. Neurother. 25, 1309–1333 (2025). 10.1080/14737175.2025.2564709 40976962

[31] Hauw J. J., Hausser-Hauw C., De Girolami U., Hasboun D. & Seilhean D. Neuropathology of sleep disorders: a review. J. Neuropathol. Exp. Neurol. 70, 243–252 (2011). 10.1097/NEN.0b013e318211488e 21412175

[32] Canto C. B., Onuki Y., Bruinsma B. & van der Werf Y. D. De Zeeuw C. I. The sleeping cerebellum. Trends Neurosci. 40, 309–323 (2017). 10.1016/j.tins.2017.03.001 28431742

[33] Rodríguez-Labrada R. Velázquez-Pérez L. Unravelling the cerebellum’s role in sleep regulation: prospects for precision sleep medicine. Sleep. Med. 140, 108797 (2026). 10.1016/j.sleep.2026.108797 41604995

[34] Dang-Vu T. T. Functional neuroimaging insights into the physiology of human sleep. Sleep 33, 1589–1603 (2010). 10.1093/sleep/33.12.1589 PMC2982729 21120121

[35] Pedroso J. L. Sleep disorders in cerebellar ataxias. Arq. Neuropsiquiatr. 69, 253–257 (2011). 10.1590/s0004-282x2011000200021 21537570

[36] Zhang L. & Zhao Z. X. Objective and subjective measures for sleep disorders. Neurosci. Bull. 23, 236–240 (2007). 10.1007/s12264-007-0035-9 PMC5550587 17687399

[37] Stephan A. M. & Siclari F. Reconsidering sleep perception in insomnia: from misperception to mismeasurement. J Sleep. Res 32 (2023). 10.1111/jsr.14028 37678561

[38] Middleton S. J. High-frequency network oscillations in cerebellar cortex. Neuron 58, 763–774 (2008). 10.1016/j.neuron.2008.03.030 PMC4852717 18549787

[39] Hobson J. A. & McCarley R. W. Spontaneous discharge rates of cat cerebellar Purkinje cells in sleep and waking. Electroencephalogr. Clin. Neurophysiol. 33, 457–469 (1972). 10.1016/0013-4694(72)90210-6 4116430

[40] Zhang L. B. Neuronal activity in the cerebellum during the sleep-wakefulness transition in mice. Neurosci. Bull. 36, 919–931 (2020). 10.1007/s12264-020-00511-9 PMC7410897 32430873

[41] Johnson E. O., Roth T. & Breslau N. The association of insomnia with anxiety disorders and depression: exploration of the direction of risk. J. Psychiatr Res. 40, 700–708 (2006). 10.1016/j.jpsychires.2006.07.008 16978649

[42] Massara M. The lateralized cerebellum: insights into motor, cognitive, and affective functioning across ages: a scoping review. J. Neurol. 272, 122 (2025). 10.1007/s00415-024-12884-2 39812809

[43] Pascual-Leone A., Rubio B., Pallardó F. & Catalá M. D. Rapid-rate transcranial magnetic stimulation of left dorsolateral prefrontal cortex in drug-resistant depression. Lancet 348, 233–237 (1996). 10.1016/s0140-6736(96)01219-6 8684201

[44] American Psychiatric Association. Diagnostic and Statistical Manual of Mental Disorders 5th edn (American Psychiatric Association, 2013).

[45] Martin J. L. & Hakim A. D. Wrist actigraphy. Chest 139, 1514–1527 (2011). 10.1378/chest.10-1872 PMC3109647 21652563

[46] Buysse D. J., Reynolds C. F., Monk T. H., Berman S. R. & Kupfer D. J. The Pittsburgh sleep quality index: a new instrument for psychiatric practice and research. Psychiatry Res. 28, 193–213 (1989). 10.1016/0165-1781(89)90047-4 2748771

[47] Lee H. C. The Zung Self-Rating Depression Scale: screening for depression among the Hong Kong Chinese elderly. J. Geriatr. Psychiatry Neurol. 7, 216–220 (1994). 10.1177/089198879400700404 7826489

[48] Zung W. W. K. A rating instrument for anxiety disorders. Psychosomatics 12, 371–379 (1971). 10.1016/S0033-3182(71)71479-0 5172928

[49] Iacono M. I. MIDA: a multimodal imaging-based detailed anatomical model of the human head and neck. PLoS One. 10, e0124126 (2015). 10.1371/journal.pone.0124126 PMC4406723 25901747

[50] McDougal R. A. Twenty years of ModelDB and beyond: building essential modeling tools for the future of neuroscience. J. Comput. Neurosci. 42, 1–10 (2017). 10.1007/s10827-016-0623-7 PMC5279891 27629590

